# Cardiovascular magnetic resonance in cardiac sarcoidosis with MR conditional pacemaker in situ

**DOI:** 10.1186/1532-429X-13-26

**Published:** 2011-05-03

**Authors:** Giovanni Quarta, Diana R Holdright, Gordon T Plant, Allan Harkness, Derek Hausenloy, Harpreet Hyare, James C Moon

**Affiliations:** 1 Department of Cardiology, The Heart Hospital, part of University College London Hospitals NHS Trust, 16-18 Westmoreland Street, London. W1G 8PH, UK; 2 Department of Cardiology, S. Andrea Hospital, University "La Sapienza", Rome, Italy; 3 National Hospital for Neurology and Neurosurgery Queen Square, University College London Hospitals NHS Trust London WC1N 3BG, UK; 4 Department of Cardiology, Colchester Hospital University NHS Foundation Trust, Colchester, UK; 5 Department of Imaging, University College London Hospitals NHS Trust, 250 Euston Road, London. NW1 2PG, UK; 6 MRC Prion Unit, Department of Neurodegenerative Diseases, UCL Institute of Neurology, Queen Square, London. WC1N 3BG, UK; 7 Department of Medicine, University College London, London, UK

## Abstract

Cardiovascular implantable electronic devices represent important limitations to magnetic resonance imaging (MRI). Recently, MRI-conditional dual chamber pacemakers and leads have become available. We describe a case of a patient with neuro-sarcoidosis presenting with diplopia and hydrocephalus requiring an MRI-conditional programmable ventriculo-peritoneal shunt, who developed complete heart block. In view of the ongoing need for neuro-imaging, MRI-conditional dual chamber pacemaker and leads were implanted. Cardiac and brain MRI were requested to guide immunosupression. Overall the scans demonstrated stable neurological disease, but confirmed cardiac sarcoid, with oedema on T2 weighted images suggesting active disease and extensive sub-endocardial late gadolinium enhancement, including the basal septum. This case illustrates why sarcoid patients who develop bradyarrhythmias should ideally have an MRI-conditional pacing system.

## Background

In the last decade, magnetic resonance imaging (MRI) has grown dramatically. At the same time, a growing number of patients receive cardiovascular implantable electronic devices (pacemaker, implantable-cardioverter defibrillators, bi-ventricular devices), a known contra-indication to MRI. It has been estimated that a patient with a cardiovascular electronic device has a 50-75% lifetime requirement for MRI, which would usually be denied [1]. A position paper from the European Heart Rhythm Association and the Working Group on Cardiovascular Magnetic Resonance of the European Society of Cardiology [1] and a scientific statement from the American Heart Association [2] on MRI in individuals with cardiovascular implantable electronic devices have been published. Recently, an MRI-conditional dual chamber pacemaker has become available and represents an important step forward to overcome one of the major limitations of MRI.

## Case presentation

**Figure 1:**
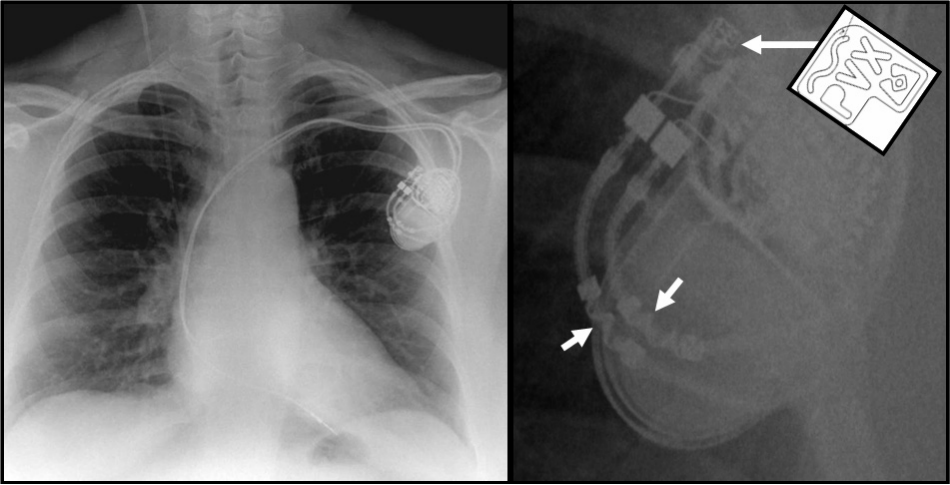
**Chest radiograph (left) and magnified image (right) of pacemaker**. White arrows show the MRI-conditional marker on the header of the can (long arrow and schematic) and similar wavy line marker on the leads (short arrows).

A 53 year old woman presented with fatigue in complete heart block. Transthoracic echocardiography was normal. Six years previously, neuro-sarcoidosis presenting with diplopia and hydrocephalus had been confirmed by meningeal biopsy, and an MRI-conditional programmable Ventriculo-Peritoneal shunt (PS Medical Strata^® ^valve, Medtronic) had been inserted. In view of her ongoing need for neuro-imaging, MRI-conditional dual chamber pacemaker and leads (Advisa DR MRI™ SureScan™ with 5086 leads, Medtronic, Figure 1) were implanted. Subsequently, in September 2010, cardiac and brain MRI were requested to guide immunosupression. Following appropriate protocols for both MRI conditional devices, interleaved pre and post contrast cardiac, brain, and orbit MRI were performed in one session at 1.5T. Prior to scanning, the pacemaker was interrogated, lead integrity checked and device switched to DOO mode at 60 bpm at 5V@1ms (from 2V@0.4ms). Post scanning, all pacemaker parameters were unaffected, and normal operation was reprogrammed. Similarly, the programmable VP shunt, whose settings alter with scanning, was reset.

**Figure 2:**
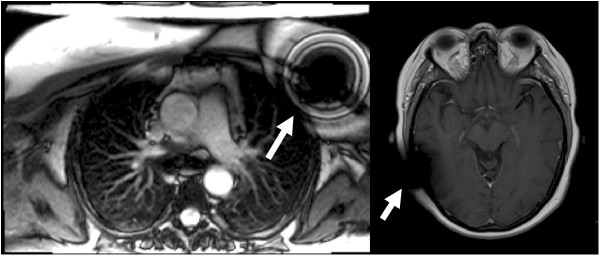
**Metallic artefact from the devices in the chest wall and skull**.

**Figure 3:**
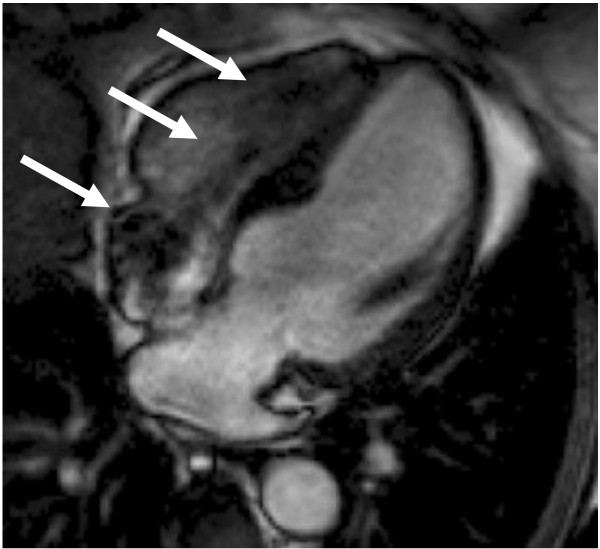
**SSFP cine 4 chamber view showing some susceptibility artefact from the pacemaker leads (white arrows)**.

**Figure 4:**
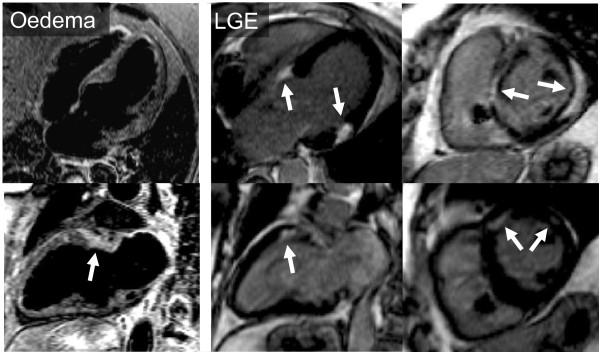
**Cardiac MRI with T2-weighted STIR images (left panel) showing oedema (arrow) and (middle and right panels) extensive patchy late gadolinium enhancement typical of sarcoid**.

Brain MRI showed susceptibility artefact associated with the shunt (Figure 2), but otherwise stable intracranial disease with scattered white matter lesions and persistent dural enhancement. Cardiac MRI showed normal LV size and systolic function with no regional wall motion abnormalities. Metallic lead artefact was minimised by switching standard SSFP cine sequence (Figure 3 and additional file 1) to spoiled gradient echo - but was not considered necessary. T2-weighted images showed basal antero-septal oedema and there was extensive sub-epicardial, sometimes transmural, basal late gadolinium enhancement (Figure 4). Overall the scans demonstrated stable neurological disease, but confirmed cardiac sarcoid, with oedema suggesting active disease.

## Conclusions

The advent of MRI-conditional devices overcomes an important limitation in disease management. Here, a multidisciplinary team approach and the use and management of two concurrent MR conditional devices [3] permitted ongoing, comprehensive assessment of multisystem sarcoidosis [4,5]. Ironically, cardiovascular MRI detected occult cardiac disease that suggests the possible future requirement for an implantable-cardioverter defibrillator. As yet, such devices are not MRI conditional. Fortunately, she has no other high risk features [6] and her device has not detected any ventricular arrhythmia. Our case illustrates why sarcoid patients who develop bradyarrhythmias should ideally have an MRI-conditional pacing system.

## Consent

Written informed consent was obtained from the patient for publication of this case report and any accompanying images. A copy of the written consent is available for review by the Editor-in-Chief of this journal.

## Conflict of interests

The authors declare that they have no competing interests.

No disclosures

## Authors' contributions

GQ and JCM have made substantial contributions to conception and design of the case, have been involved in drafting the manuscript and revising it critically for important intellectual content and have given final approval of the version to be published.

DRH, GTP, AH, DH and HH have made substantial contributions to conception and design of the case, have been involved in revising the manuscript critically for important intellectual content and have given final approval of the version to be published.

## Supplementary Material

Additional file 1 **Top: SSFP gradient echo cine (left) and spoiled gradient echo (right) cine four chamber views**. SSFP images are more susceptible to artefacts from pacemaker leads, but image quality is good. Bottom: SSFP-GRE short axis views, showing no regional wall motion abnormalities. Click here for file
